# Green Synthesis of Gold, Iron and Selenium Nanoparticles Using Phytoconstituents: Preliminary Evaluation of Antioxidant and Biocompatibility Potential

**DOI:** 10.3390/molecules27041334

**Published:** 2022-02-16

**Authors:** Abeer Jabra Shnoudeh, Lana Qadumii, Malek Zihlif, Hamzeh J. Al-Ameer, Ruba Anwar Salou, Abdulmutalleb Yousef Jaber, Islam Hamad

**Affiliations:** 1 Department of Pharmaceutical Sciences, Faculty of Pharmacy, Philadelphia University, Amman 19392, Jordan; ashnoudeh@philadelphia.edu.jo (A.J.S.); ajaber@philadelphia.edu.jo (A.Y.J.); 2 Department of Basic Sciences, Faculty of Science, Philadelphia University, Amman 19392, Jordan; lqadumii@philadelphia.edu.jo (L.Q.); rsalou@philadelphia.edu.jo (R.A.S.); 3 Department of Pharmacology, School of Medicine, The University of Jordan, Amman 11942, Jordan; m.zihlif@ju.edu.jo (M.Z.); h.alameer@aum.edu.jo (H.J.A.-A.); 4 Department of Biology and Biotechnology, Faculty of Science, American University of Madaba, Madaba 11821, Jordan; 5 Department of Pharmacy, Faculty of Health Sciences, American University of Madaba, Madaba 11821, Jordan

**Keywords:** gold-nanoparticles, iron-nanoparticles, selenium-nanoparticles, antioxidant potential, biocompatibility, green synthesis, *Punica grantum*, Ephedra, Pistachio leaves

## Abstract

This study aimed at fabricating gold (Au), iron (Fe) and selenium (Se) nanoparticles (NPs) using various natural plant extracts from the Fertile Crescent area and evaluating their potential application as antioxidant and biocompatible agents to be used in the pharmaceutical field, especially in drug delivery. The Au-NPs were synthesized using *Ephedra alata* and *Pistacia lentiscus* extracts, whereas the Fe-NPs and Se-NPs were synthesized using peel, fruit and seed extracts of *Punica granatum*. The phytofabricated NPs were characterized by the UV-visible spectroscopy, scanning electron microscope, Fourier transform infrared spectroscopy, X-ray diffraction (XRD) and energy-dispersive X-ray (EDS) spectroscopy. Scanning electron microscope technique showed that the synthesized NPs surface was spherical, and the particle size analysis confirmed a particle size of 50 nm. The crystalline nature of the NPs was confirmed by the XRD analysis. All synthesized NPs were found to be biocompatible in the fibroblast and human erythroleukemic cell lines. Se-NPs showed a dose-dependent antitumor activity as evidenced from the experimental results with breast cancer (MCF-7) cells. A dose-dependent, free-radical scavenging effect of the Au-NPs and Se-NPs was observed in the DPPH (2,2-Diphenyl-1-picrylhydrazyl) assay, with the highest effect recorded for Au-NPs.

## 1. Introduction

Nanotechnology is considered among the most emerging branches of science during the last decade. Research interest in this field, especially in the field of nanoparticles, NPs (particle sizes in the range of 1 to 100 nm) are gaining a high interest in this field. The applications of these NPs have been extended to the treatment of various complex ailments and to other fields, such as biomedical and food industries [1,2,3,4]. Simultaneous research in metallic NPs has also emerged for their evolving applicability in various industrial fields of biotechnology [5], electronic [6] drug delivery [7,8] and targeted drug delivery [9] in the pharmaceutical field [10], gene delivery [11], and food industry [12].

In the last few years, scientists paid great attention to the synthesis of metal NPs and their applications. Numerous chemical, physical and biological methods have been used in the synthesis of NPs. Biochemical methods used for the synthesis of metallic NPs were considered to be economic and safe [13,14]. A wide range of biomacromolecules have been explored in the preparation of nanomaterials, where these agents acted as potential biocatalysts. Additionally, these biomacromolecules could also serve as natural stabilizers for the NPs [15]. In the synthesis process of metal NPs, components from plants or parts of plants, algae, fungus, or bacteria can be incorporated. The use of phytomolecules-based synthesis of NPs is of great interest as this process is relatively easy, rapid and does not require a special reaction environment [16]. For this reason, to utilize the benefit of the phytochemicals, the extracts of *Punica granatum* (pomegranate) peel, seeds and fruits [17], ephedra [18] and pistachio leaves [19] were used in the synthesis process in this study.

Gold nanoparticles Au-NPs are extensively used in the biomedical field and have been introduced in drug delivery systems to transport gene materials and drugs. Besides, Au-NPs have been used in biological and chemical sensing and diagnosis [20,21,22]. Similarly, the application of iron nanoparticles Fe-NPs has been extended to various surface treatments and biomedical applications due to their superparamagnetic properties and compatibility with the biological system, as well as their high safety profile [23]. Alternatively, the semiconductor material selenium has been introduced in the biomedical field as an essential trace mineral that might prevent cellular damage, maintain the thyroid gland, help proper functioning of the immune system, and provide antimicrobial and anti-carcinogenic activity [24,25]. Because of a wide range of applications of Au-NPs, Fe-NPs, and selenium nanoparticles Se-NPs, the present research was dedicated to the synthetization of the respective NPs using phytoconstituents as natural reducing agents in the chemical reaction to produce the required NPs.

In this study, green synthesis using plant’s extracts, which is a biological approach used in NPs synthesis, was chosen for its superiority over physical and chemical approaches for being environmentally friendly and less toxic [24]. The green chemistry has been used for the synthesis of Au-NPs by the reduction of selected oxidizing agents using different plants, such as ephedra (*Ephedra alata*) and pistachio extracts (*Pistacia* leaves). However, Fe-NPs and Se-NPs were synthesized from the extracts of *Punica granatum* peel, seeds, and fruit. The plants were chosen according to availability and phyto constituents; *Punica granatum* peel [26], ephedra [27] and pistachio leaves [28] extracts share features of having flavonoids and phenolic compounds. Characterization of the synthesized NPs was carried out using various methods including visible spectroscopy, Fourier transform infrared spectroscopy (FTIR), X-ray crystallography (XRD), scanning electron microscopy (SEM) and energy-dispersive X-ray spectroscopy (EDX). Additionally, in vitro evaluations of antioxidant efficacy, and biocompatibility study in three different cell lines were performed to establish the applicability of the formulated NPs.

## 2. Results and Discussion

Chemical synthesis of NPs may produce hazardous toxic manifestations because of the presence of toxic organic residues within the product. The biogenic synthesis using natural products are nowadays adopted due to their simplicity and ease of preparation. Furthermore, NPs synthesized using biogenic methods are free from any toxic residues and thus are free of any hazardous matters [15,29]. There is some evidence available in the literature on the low toxic profile of the NPs synthesized through green synthesis [30,31]. Keeping this in view, the green synthesis approach was adopted in this study to synthesize Au-, Fe- and Se-NPs.

**Figure 1:**
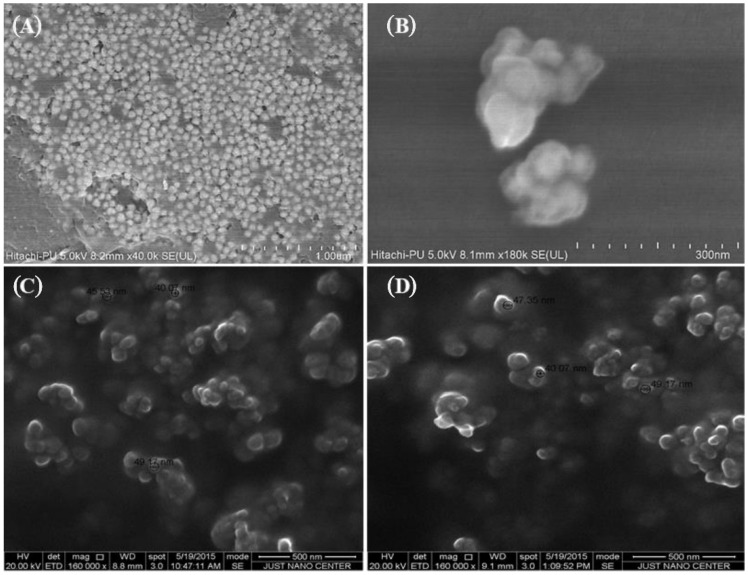
Scanning electron micrographs of prepared NPs. Representative images of (**A**) gold NPs prepared from HAuCl_4_ in the presence of ephedra extract (Scale bar 1000 nm), (**B**) gold NPs prepared from HAuCl_4_ in the presence pistachio leaf extracts (Scale bar 300 nm), (**C**) iron NPs prepared using ferrous sulfate as iron precursor, and *Punica granatum* peel extract as the reducing agent and stabilizer (Scale bar 500 nm), (**D**) selenium NPs prepared using selenous acid in the presence of *Punica granatum* peel extract as the reductant (Scale bar 500 nm).

Particle size of NPs affects their properties and applications as well as their toxicity [32,33]. The particle size of the Au-NPs obtained from the ZetaSizer were 39.16 ± 1.28 nm (ephedra extract Figure S1A) and 62.21 ± 3.42 nm (pistachio extract), while the size was comparable for Fe-NPs (44.15 ± 2.76 nm) (Figure S1B) and Se-NPs (47.35 ± 1.23 nm) (Figure S1C) prepared using a *Punica granatum* extract. The SEM images of the Au-NPs, Fe-NPs, and Se-NPs showed the spherical nature of the particles (Figure 1 and Figure S2).

During NPs synthesis, extracts from the plant component was simply mixed with the aqueous solution of the respective metal salt at room temperature. With this approach, different parts of plants could be used as reducing agents in the synthesis of metal NPs [34,35]. Basically, the metal NPs are formed by the process of reduction of the metal ions in the metal salts by means of the natural reducing agents. The particles formed as a result of the reduction process are generally aggregated and then form small clusters that grow to form the NPs. Then, the plant materials form a coat over the NPs that prevent more aggregation. These findings are reflected by the dynamic light scattering results. However, agglomerations of the metallic NPs have been observed in the SEM images, which might be due to the increased temperature of the formulation during analysis, or due to the “coffee-stain effect” [36,37]. The results obtained in this study are in agreement with the report by Kulkarni and Muddapur [38].

### 2.1. Characterization of Au-NPs

**Figure 2:**
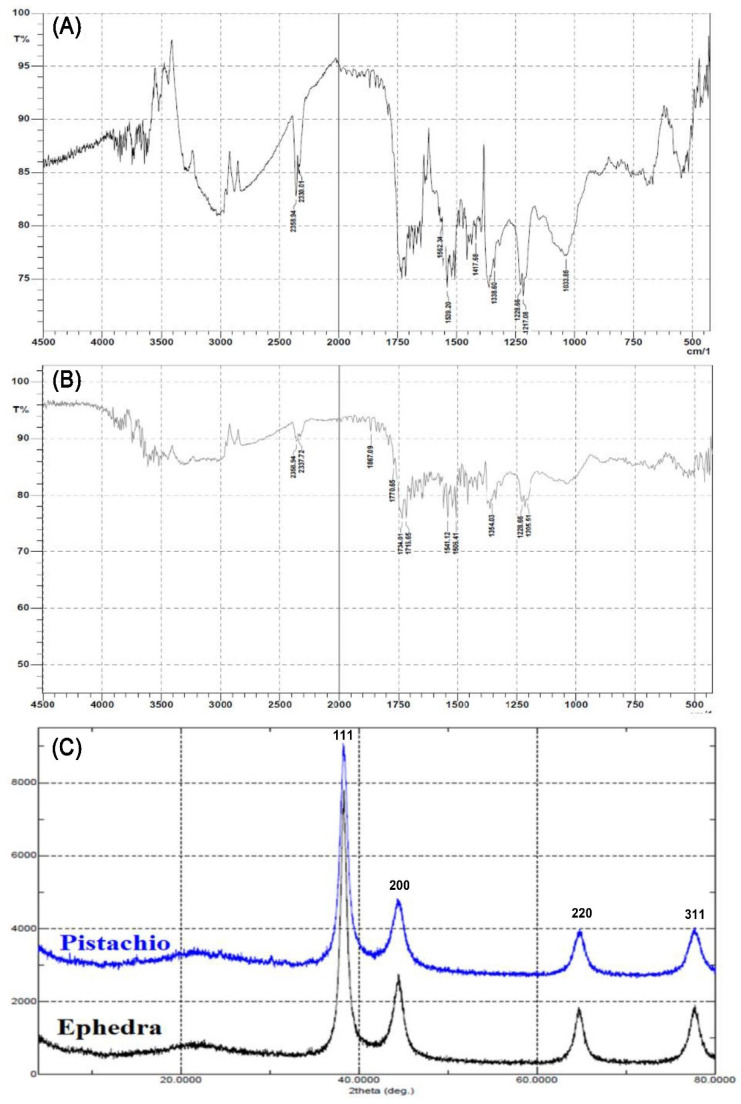
Characteristics of gold NPs. (**A**) Representative FTIR spectroscopic image of gold NPs prepared from HAuCl_4_ in the presence of ephedra extract, (**B**) FTIR spectrum of gold NPs prepared from HAuCl_4_ in the presence of pistachio leaf extract, and (**C**) X-ray diffraction overlay of gold NPs prepared with ephedra and pistachio leaf extracts.

FTIR spectrum of the synthesized Au-NPs was recorded to investigate the functional groups present. As seen in Figure 2a,b, peaks in the range of 3800–3600 cm^–1^ could be attributed to the phenolic groups of the ephedra and pistachio leaf, whereas peaks at 1715, 1506, 1354 and 1218 cm^–1^ would be attributed to carbonyl C=O stretch, C=C aromatic, CH_3_C–H bending in alkyls, and R–O–R (ether), respectively. When comparing the FTIR of *Ephedra alata* extract [39] with the FTIR of Au-NP synthesized from ephedra, some differences were observed at different peaks indicating the involvement of different functional groups in the formation of Au-NP. Also, some differences are observed when comparing the FTIR of the pistachio leaf extract [40] with the FTIR for Au-NP synthesized from pistachio leaf extract for peaks, which indicates the involvement of the OH groups present the phytoconstituents in the formation of Au-NP.

Furthermore, the Au-NPs were characterized by XRD analysis, where the XRD pattern of the Au-NPs is presented in Figure 2c. The major Bragg reflection at 2θ values was observed at 38.11°, 42.34°, 62.43°, 78.21°, which correspond to the Bragg reflections (111), (200), (220) and (311) of the planes of crystalline Au. The XRD data was compared to ICDD/PDF 00-004-0784.

### 2.2. Characterization of Fe-NPs

In this study, Fe-NPs were synthesized successfully from the *Punica granatum* peel, juice and seeds, by using different oxidizing iron salts solutions at different pH values. The best results were achieved from the peel and juice of the *Punica granatum* using FeCl_3_ and FeSO_4_ in an acidic medium. These observations were also seen by Mahdavi et al. [41]. It was noticed that decreasing the pH resulted in a better involvement of OH group of the plant extract in the reduction process.

The spectrum recorded by UV-visible spectrophotometry for the Fe-NPs produced showed absorption peaks at 402 and 416 nm. The color of the reaction mixture was changed to dark violet, which is also an indication of the formation of Fe-NPs. Furthermore, acidic medium was shown to have a better environment for synthesizing Fe-NPs and this is in agreement with the results of Makarov et al., where they indicated that Fe-NPs showed better stability when the synthesis was carried out in an acidic medium [42].

**Figure 3:**
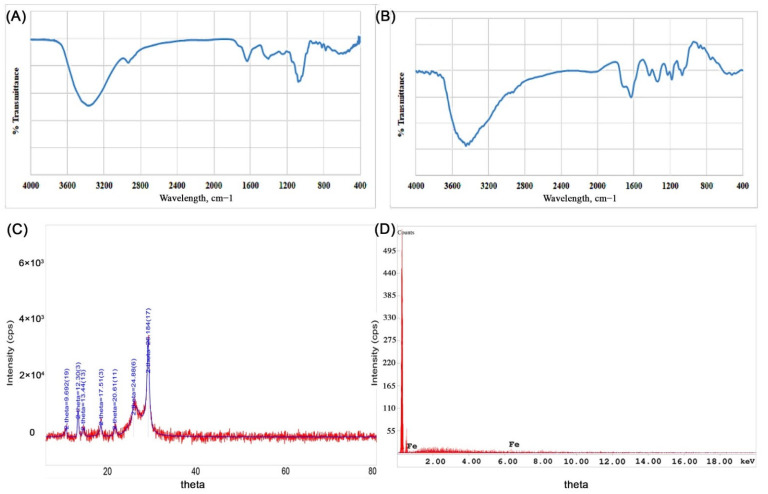
Characteristics of iron NPs. (**A**) Representative FTIR spectrum of *Punica granatum* peel extract, (**B**) FTIR spectrum of Fe-NPs prepared using ferrous sulfate as iron precursor and *Punica granatum* peel extract as reducing agent and stabilizer, (**C**) X-ray diffraction pattern of iron NPs prepared using ferrous sulfate as iron precursor and *Punica granatum* peel extract as reducing agent and stabilizer, (**D**) energy-dispersive X-ray analysis of iron NPs prepared using ferrous sulfate as iron precursor and *Punica granatum* peel extract as reducing agent and stabilizer.

The FTIR spectra of the synthesized Fe-NPs were used to investigate the influence of the extracts employed in the synthesis of the NPs. As seen in Figure 3a,b, peaks at 3543, 1739, 1324 and 1188 cm^–1^ correspond to the presence of carbonyls; carbonyl C=O stretching, CH_3_C-H bending in alkyls, and R-O-R in ether, respectively. Also, the broad peak between 3200–3500 cm^–1^ indicates to the presence of amino group, -NH_2_, which was also present in the FTIR of *Punica granatum* peel. Some differences were observed when comparing the FTIR of the *Punica granatum* peel and the FTIR of Fe-NP synthesized from *Punica granatum* peel at the functional group peaks (Figure 3a,b), which confirms the involvement of the functional groups in the phytoconstituents in the synthesis of Fe-NP.

The nature of Fe-NPs was also assessed by XRD analysis, where the XRD pattern of the Fe-NPs is presented in Figure 3c. The major Bragg reflection 2θ values were observed at 9.66°, 12.30°, 13.44°, 17.51°, 20.61°, 24.88°, and 28.18°, which might correspond to the planes of the crystalline Fe. This may be attributed to the lower angle shift in the XRD pattern due to planar stress, or to a change in stoichiometry, or to the presence of mixed iron oxides NPs. However, Holder and Schack [43] explained is the difficulty of phase identification by XRD for some systems, as is the case for nanoscale materials. Moreover, iron oxides Fe_3_O_4_ and Fe_2_O_3_, which might be present with the Fe-NPs, have broad peaks that cannot be distinguished. On the other hand, *Punica granatum* (pomegranate) chemistry is complex, as it contains around 124 different phytoconstituents [44]. The complete understanding of the angle shift in XRD pattern remains unclear and needs further investigations in the future. The Fe-NPs XRD data were compared to the ICDD Powder Diffraction file number 00-006-0696.

EDX spectrum of the Fe-NPs, which is represented in Figure 3d, shows the presence of Fe-NP with an elemental composition of 100% Fe. Figure S3 represents the EDX spectrum of the Fe-NPs formulated using different iron salts and *Punica granatum* samples, representing a similar pattern of Fe-NPs. 

### 2.3. Characterization of Se-NPs

The synthesis of Se-NPs using *Punica granatum* peel, juice and seeds, from different selenium salt solutions, revealed that the peels and juice extracts provide satisfactory results, where the results of these two extracts were almost similar in pattern. It has been observed that the acidic medium provides a better environment for the synthesis of Se-NPs.

**Figure 4:**
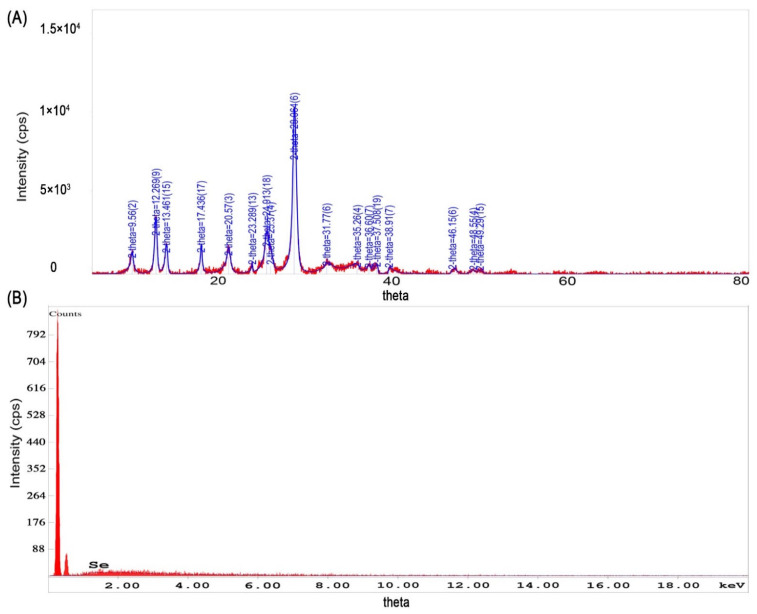
Characteristics of selenium NPs. (**A**) X-ray diffraction pattern of selenium NPs prepared from selenous acid in the presence of *Punica granatum* peel extract, (**B**) energy-dispersive X-ray analysis of selenium NPs prepared from selenous acid and *Punica granatum* peel extract as the reducing agent.

FTIR spectra of Se-NPs did not show clear evidence of the presence of functional groups. However, there were several peaks displayed in the XRD pattern as represented in Figure 4a. The crystallinity of the Se-NPs is evidenced by the sharp Bragg’s peaks at 8.56°, 12.26°, 13.46°, 17.43°, 20.57°, 25.37°, and 28.06°. However, broad peaks have been observed at 31.77°, 35.26°, 36.6°,37.5°, 3.69°, 38.91°, 46.15°, 48.55°, and 49.29° which could be, as mentioned above, due to the same reasons that caused the lower angle shift in the XRD pattern for the iron oxide NPs. Moreover, the selenous acid could have been retained with the formed selenium NP, which could have bound to the phytoconstituents of the *Punica granatum* and caused this angle shift. This can be confirmed by the presence of similar angles in the iron NP XRD. The Se-NPs XRD data were compared to ICDD Powder Diffraction file number 00-051-1389.

The EDX analysis report showed the presence of Se element of 100 Wt%, and the presence of the initial two peaks could be due to C and O, Figure 4b, which comes from the support grid and film; however, the percentage of these two components were very low. Similarly, the EDX spectrum of the Se-NPs formulated using different selenium oxidizing reagents with *Punica granatum* extracts, showed similar pattern which is represented in Figure S4; however, the presence of element Se less than 100 Wt%. 

### 2.4. Antioxidant Potential of the NPs

**Figure 5:**
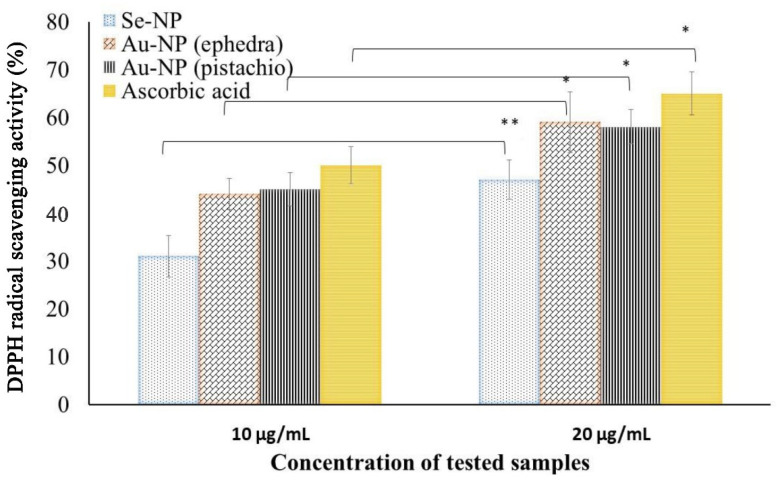
DPPH (2,2-diphenyl-1-picrylhydrazyl) free radical scavenging antioxidant potential of the phytosynthesized gold (Au-NPs) and selenium (Se-NP) NPs. All values are presented as the mean ± standard deviation (*n* = 3); * *p* < 0.05 ** *p* < 0.01.

The antioxidant activity of the synthesized NPs was assessed by DPPH free radical scavenging activity. Amongst the three synthesized NPs, Se-NPs and Au-NPs had shown significant antioxidant activity at the concentration range of the NPs tested. Fe-NPs did not show any free radical scavenging potential within the range assessed in the current experimental conditions. The potential antioxidant activity is represented in Figure 5, which exhibits scavenging potential activity proportional to the concentration of the NPs.

The antioxidant efficacy of the Au-NPs obtained the following reactions with ephedra and pistachio leaf extract was found to be close to ascorbic acid. Although, the antioxidant activity of the phytosynthesized NPs could be explained by the adsorption of phytoconstituents from the materials used in the synthesis process by the NP. The antioxidant activity of the NPs increases (*p* < 0.05) significantly at the higher dose used (20 µg/mL) when compared to the lower dose used (10 µg/mL). Our findings on the antioxidant activity of Se-NP are congruent with the findings in the literature for the synthesized Se-NPs using *Emblica officinalis* fruit extract [25]. The results of Se-NPs on antioxidant activity are similar to the reports on green-synthesized Se-NPs in a similar study [45]. Similarly, the findings of Au-NPs in this study were in line with the findings of Oueslati et al. [46].

### 2.5. Cytotoxicity Outcomes of the Synthesized NPs

**Figure 6:**
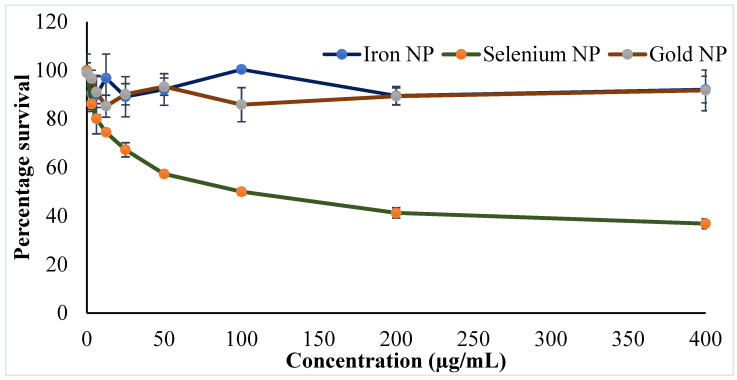
Cytotoxicity effect in terms of percentage survival of the breast cancer cell line (MCF-7) following treatment of synthesized gold (Au-NP), iron (Fe-NP), selenium (Se-NP) NPs. All values are presented as the mean ± standard deviation (*n* = 3); * *p* < 0.05 vs. positive control group.

Progress of nanotechnology has moved to develop personalized medicine with improved efficacy and safety. Simultaneous investigations of inorganic NPs concurrently with polymeric and lipid NPs have shown a successful chapter in cancer treatment [47,48]. Cytotoxicity studies using cell lines are a good choice to predict the adverse effects of NPs in humans and in the absence of animals [30,49]. An evaluation of the cytotoxicity of the developed inorganic NPs was investigated in cancerous and normal cells. Amongst the NPs, Se-NPs showed to induce cytotoxicity against the MCF-7 cell line, whereas other two NPs (Fe-NPs and Au-NPs) did not produce any cytotoxic potential in the current experimental conditions. Findings of anticancer activity by the Se-NPs provide hope in fighting against the shocking numbers of breast cancer cases worldwide. The findings on the survival of MCF-7 cells following 24 h of NP treatment were presented in Figure 6. The cytotoxic potential of the Se-NPs was found to be dose dependent, where the increase in dose significantly decreases (*p* < 0.05) the survival of the MCF-7 cell lines. Furthermore, the effect of the phytoconstituents at the concentration used in the preparation of the NPs was checked for cytotoxic potential, and the results depicted no cytotoxicity of the phytoconstituents to the MCF-7 cell line (Table S1). Therefore, it could be concluded that the anticancer activity of Se-NPs might be ascribed to the NPs, not to the phytoconstituents incorporated in the preparation method.

It is believed that the Se-NPs are internalized within the cells via receptor-mediated endocytosis, which in the acidic medium of the cancerous cell leads to the pro-oxidant conversion of Se-NPs. Thus, the generated free radicals may damage the mitochondrial membrane, leakage of various proteins, followed by caspase-mediated apoptosis. On the other hand, the Se-NPs create cellular stress on the endoplasmic reticulum, which activates multiple molecular paths, perhaps via Wnt/β-catenin, PI3K/Akt/mTOR, MAPK/Erk, and NFκB, to ultimately induce the apoptosis of the cancer cells [47]. These mechanisms have established the efficacy of the phytosynthesized Se-NPs against breast cancer, where the IC_50_ of the synthesized Se NPs was established as 100 µg, whereas the Fe and Au NPs did not show a significant activity up to 400 μg. Further, prevention of cancer cell growth by those agents with anticancer potential is facilitated by the arrest of the cell cycle or the induction of apoptosis, or even a combination of both the mechanisms. Different researchers such as Yang et al. and Zhu et al. had performed cytometric analysis to determine the underlying mechanism of the induced cell death of the cancer cells by the action of Se-NPs. Studies revealed that Se-NPs could not possess significant changes in the cell cycle arrest [50], thus, the major mechanism of cancer cell death by the Se-NPs could be due to cellular apoptosis. Yang et al. reported a similar finding for the explanation of the anticancer potential of Se-NPs [51]. The research explanation by Zheng et al. revealed the apoptotic potential of the Se-NPs on cancer cells by the process of nuclear condensation and the generation of apoptotic bodies. Furthermore, analysis by the authors reported the cleavage of PARP and the activation of caspase-3 towards the induction of such apoptosis [50].

Alternatively, the synthesized NPs were found to be safe in normal cell lines, as depicted from the results on fibroblast cell, indicating that the formulations are safe to the normal cells whenever administered; however, further studies are necessary to establish the safety of these inorganic NPs.

The biocompatible characteristics of the NPs are also observed in experimented K562 cell lines, where no inhibition of growth was recorded. The entire synthesized metal-NPs showed no cytotoxicity up to the dose of 400 µg when using fibroblasts cells and K562 cell lines.

The role of Au-NPs in the cancer microenvironment has been explored to establish as a diagnostic agent. Simultaneously, regulated photothermal activity of Au-NPs has also been progressed to develop antitumor effect [52]. Thus, the safety of the Au-NPs towards the normal cells could further be projected for a theranostic approach in the future.

## 3. Materials and Methods

### 3.1. Materials

*Punica granatum* fruits were obtained from local farm in Salt Jordan, *Pistacia* leaves were obtained from a pistachio nut tree planted at the campus of Royal Scientific Society, El Hassan Science City, Jordan and the *Ephedra alata* was obtained from Jenin in Palestine. Hydrogen tetrachloroaurate (III) hydrate (HAuCl_4_ ·3H_2_O), selenous acid (H_2_SeO_3_), selenium tetrachloride (SeCl_4_), iron (III) perchlorate (Fe(ClO_4_)_3_), 2,2-diphenyl-1-picrylhydrazyl (DPPH), 3-(4,5-dimethylthiazol-2-yl)-2,5-diphenyltetrazolium bromide) (MTT), iron chloride (FeCl_3_·6H_2_O, 99.8%), ferrous sulfate (FeSO_4_), Chitosan, and Gum Arabic were purchased from Sigma Aldrich, St. Louis, MO, USA. The cell culture media, Dulbecco’s modified Eagle high glucose media (DMEM), were obtained from Roswell Park Memorial Institute (RPMI) 1640 media (Euroclone SPA, Italy). Fetal bovine serum (FBS), L-glutamine, streptomycin, and penicillin were procured from HyClone, Logan, UT, USA. The deionized water used in different experiments was obtained from MilliQ^®^ purified water system (Millipore, Bedford, MA, USA). Other chemicals used in this study were of analytical grade.

### 3.2. Green Synthesis of NPs

NPs were prepared by green synthesis, using a simple reduction method, where the materials from plants are used as a reducing agent. The detailed methodology of individual NPs is described in the following subsections.

#### 3.2.1. Synthesis of Gold NPs

Preparation of Au-NPs was performed (*n* = 3) following the method described in the literature [18] with minor modifications using aqueous extracts of ephedra and pistachio leaves as reducing and capping agents. Briefly, 1 mL of the extracts was added to the oxidizing agent of gold (0.005 M HAuCl_4_) at pH 7.0. The concentration of HAuCl_4_ was 0.005 M, and the reaction was done at the ratio of 1:1.5 (HAuCl_4_: extract).

The reaction was carried out at room temperature in an aqueous environment. Changes in the color of the mixture from yellow to pink and then to red was considered as an indication of reaction process. The formation of NPs was monitored by UV-Vis absorption spectrometer (Optima, Tokyo, Japan) using wavelengths of 560, 570, 590, and 610 nm. Upon completion of the reaction, the mixtures were centrifuged (Hermle Z233 MK-2; Hermle, Wehingen, Germany) at 5000 rpm for 10 min to separate the NPs. The collected NPs were then re-suspended in distilled water to remove any impurities. The method was repeated thrice and finally dried in vacuum at 30 °C.

#### 3.2.2. Synthesis of Iron Oxide NPs

Fe-NPs were synthesized (*n* = 3) from three different iron (III) oxidizing agents, namely FeCl_3_, FeSO_4_ and Fe(ClO_4_)_3_, using a 75% ethanol extract of *Punica granatum* peel, seeds and fruit as reducing and capping agents [53,54]. Oxidizing reagents were prepared by mixing 0.1 M of the oxidizing agent with 1% chitosan, 1 M NaCl and 0.56 M glucose. The reaction was performed at a ratio of 1:1 (extract:oxidizing agent solution) at room temperature and at different pH, and was monitored at different wavelengths. Specifically, for FeCl_3_: pH 1.3; wavelengths (λ) 402 and 415 nm, FeSO_4_: pH 2.83; wavelength (λ) 283 nm, and Fe(ClO_4_)_3_: pH 3; wavelengths (λ) 402 and 416 nm. The change in color of the mixture to black represented the formation of Fe-NPs. Further, the product was processed as described in Section 3.2.1.

#### 3.2.3. Synthesis of Selenium Oxide NPs

Se-NPs were synthesized (*n* = 3) from two different oxidizing agents, H_2_SeO_3_ and SeCl_4_, using the same extracts used in the preparation of the Fe-NPs as reducing and capping agents. The reaction was carried out in an acidic medium of pH of 1.22 at room temperature and the product was monitored at wavelengths of 210 and 218 nm. Oxidizing reagents were prepared by mixing 0.1 M of the oxidizing agent with, 1% gum Arabic, 1 M NaCl and 0.56 M glucose and the reaction was performed at a volume ratio of 1:1 (plant extract:oxidizing reagent solution). The change in color of the reaction mixture to red indicated the formation of Se-NPs [55]. Furthermore, the product was processed as described in the previous Section 3.2.1.

### 3.3. Characterization of the Synthesized NPs

#### 3.3.1. Determination of Particle Size of the NPs

The hydrodynamic size of the synthesized NPs was measured by focusing a monochromatic light through the suspended samples in aqueous media. The distribution of sizes of the NPs was measured using ZetaSizer (Nano ZS, Malvern Instrument, Malvern, UK) at room temperature [56].

#### 3.3.2. Determination of Morphology and Elemental Analysis of NPs

The shape, surface morphology, and size of NPs were determined by scanning electron microscopy (SEM) (Hitachi Ltd., Tokyo, Japan). Before loading the samples into the sample holder, NPs suspensions were air-dried, then particles were gold-coated in a vacuum using a sputter coater [57]. Images were obtained by different magnifications at 20 kV.

Further, the analysis was extended to determine the elemental compositions of NPs by energy-dispersive X-ray analysis (EDX) at 20 kV using Quanta FEG 450 (FEI Company, Hillsboro, OR, USA). NPs suspended in ethanol were placed on the sample-loading grid, which was then evenly dried before elemental analysis.

#### 3.3.3. Fourier Transform Infrared (FTIR) Spectroscopic Analysis

The presence of various reducing and stabilizing functional groups as capping agents on the respective NPs was analyzed by FTIR spectroscopy. The dried NP samples were grounded homogenously to obtain the spectra within the wave number range of 400–4000 cm^–1^ against the potassium bromide background [58] using FTIR spectrophotometer (IRAffinity-1S, Shimadzu, Tokyo, Japan).

#### 3.3.4. X-ray Diffraction (XRD) Analysis

The XRD analysis of the formulated metal NPs was performed by the Rigaku Ultima IV X-ray diffractometer (Rigaku Corp., Akishima, Tokyo, Japan). The instrument is attached to a copper (Cu) anode, which was the X-ray source at a λ of 1.5406 Å. The instrument was operated at 40 mA and 40 kV to record the results over a 2θ range up to 80° according to the literature [59].

### 3.4. Anti-Oxidant Activity

The free radical scavenging potential of the developed NPs was analyzed by the radical scavenging activity of DPPH following the reported methods [60,61]. Briefly, NPs at concentrations of 10 and 20 µg/mL were mixed with 0.5 mL of 250 μM methanolic DPPH solution, 1 mL acetate buffer and the final volume was made up to 3 mL using methanol. Later, the reaction mixture was shaken and kept in a dark environment undisturbed at 25 ± 2 °C for 30 min and the absorbance was measured at 517 nm using the UV-visible spectrophotometer. The antioxidant activity of NPs was calculated by determining the decrease in the absorbance at different concentrations following the mentioned equation.

$$\mathrm{DPPH}\;\mathrm{radical}\;\mathrm{scavenging}\;\mathrm{activity}\;=\frac{\left(1-\mathrm{absorbance}\;\mathrm{of}\;\mathrm{the}\;\mathrm{test}\;\mathrm{sample}\right)}{\left(\mathrm{Absorbance}\;\mathrm{of}\;\mathrm{control}\;\mathrm{sample}\right)}\times 100$$

### 3.5. Biocompatibility Assay

The biocompatibility of the formulated NPs was evaluated using an in vitro cell line through cytotoxicity analysis.

#### 3.5.1. Cell Culture and Treatment

In order to perform the MTT assay of the NPs, three different cell lines namely human breast adenocarcinoma cancer cell line (MCF-7), erythroleukemia cell line (K562) and non-cancerous (normal) human fibroblasts cell line were chosen. The cell strains were obtained from the American Type Cultural Collection (ATCC; Manassas, VA, USA) available in the cell bank of Tissue Culture Unit. The fibroblast cells were sub-cultured in DMEM media and the MCF-7 and K562 cells were grown in RPMI 1640 medium. Both the media were supplemented with FBS (10% *v*/*v*), 1% L-glutamine (2.0 mM), streptomycin (100 µg/mL) and penicillin (100 U/mL). Prior to experimentation, cells were grown to 80% confluence in 75 cm^2^ cell culture flasks (Membrane Solutions; North Bend, WA, USA). The cells were incubated in a humidified environment of 37 °C and 5% CO_2_/ 95% air (normoxic conditions) in a cell culture incubator (Nuaire, Plymouth, MN, USA). The cultured cells were counted in an automated cell counter and 15 × 10^4^ cells of each were seeded into the 96-well cell-cultured plate. The cells were allowed to attach to the well surface; thus the plates were kept in the incubator for 24 h. The stock preparations of the different NPs were prepared in DMSO, where the working standards samples were prepared in DMEM media maintaining the DMSO concentration of <0.01%.

#### 3.5.2. Cytotoxicity Assay

MTT-assay was used to determine the viability of NP-treated cells. MTT is a yellow dye, which is reduced by cellular enzymes to the blue colored product, formazan. This enzymatic transformation is only possible in viable cells, thus the amount of blue formazan produced is directly proportional to the number of viable cells [62].

Cell Titer non-radioactive cell proliferation assay kit^®^ (Promega; Madison, WI, USA) was used to examine the cytotoxicity of NPs on non-cancerous and cancerous cell lines. The 96-well plate with attached cells was treated with the working standard solutions of the NPs (200 μL) by replacing the media inside the wells. Treated cells with different concentrations of NPs were further incubated for 24 h in the incubator. Following incubation of the cells, the free NPs in the well were removed followed by incubation of the treated cells with MTT solution (5% *w*/*v* in phosphate buffer saline) for 4 h. Finally, removal of supernatant MTT solution was done and the cells were exposed to a mixture of DMSO, acetic acid and sodium dodecyl sulfate (99.4 mL: 0.6 mL:10 g) (150 μL) to solubilize the formed formazan crystals. The generated amount of blue formazan was determined using microplate reader (Tecan, Salzburg, Austria) at 570 nm. The wells without cells served as a negative control, and their absorbance was subtracted from the other results. Untreated cells served as positive control. The results were calculated as the percentage of viability in relation to the untreated cells [63].

### 3.6. Statistics

Statistical analysis was performed by using GraphPad Prism software version 8.4.3 (GraphPad Software, Inc., San Diego, CA, USA) to measure the IC_50s_ of each tested NPs through a nonlinear regression log(inhibitor) vs. response equation. Values are expressed as the mean ± SD, and data were analyzed by using one-way ANOVA, followed by Tukey’s multiple comparison test, with the level of significance set at *p* < 0.05. The values are the means of triplicate experiments.

## 4. Conclusions

In summary, the three NPs were synthesized by the green synthesis method, where Au-NPs were synthesized using ephedra and pistachio extracts, and Fe-NP and Se-NP were synthesized using peel, seeds, and fruit extracts of *Punica granatum*. The synthesized NPs were further confirmed by different characterization procedures; FTIR, XRD, EDX and SEM. The metal-NPs produced were approximately 50 nm in size; an acidic medium was shown to have a better environment for synthesizing iron and selenium NPs, whereas a neutral medium was found to be the best for the synthesis of Au-NPs. The formulated NPs did not inhibit the cell proliferation of fibroblast and human erythroleukemic cell lines, exhibiting biocompatible nature of the NPs. Conversely, the Se-NPs showed dose-dependent cytotoxicity against MCF-7 cell lines, which could be an indication for exploring these NPs in the treatment of breast cancer. Furthermore, the dose-dependent antioxidant efficacy of the NPs containing phytoconstituents opens up avenues for the widespread application of these NPs in the pharmaceutical, biomedical and food industries.

## Data Availability

The data presented in this study are contained within the article.

## Supplementary Materials

The following are available online, Figure S1: histograms obtained from the ZetaSizer for the (A) gold NPs prepared using HAUCl_4_ and ephedra extract, (B) iron NPs prepared using ferrous sulfate as iron precursor and *Punica granatum* peel extract as reducing agent and stabilizer and (C) selenium NPs prepared using selenous acid and *Punica granatum* peel extract, Figure S2: scanning electron micrographs of prepared NPs. Representative images (A) iron NPs prepared using ferrous sulfate as iron precursor and *Punica granatum* peel extract as reducing agent and stabilizer (Scale bar 500 nm), (B) iron NPs prepared using iron (III) perchlorate as iron precursor and *Punica granatum* peel extract as reducing agent and stabilizer, (C) selenium NPs prepared using selenium tetrachloride and *Punica granatum* seed extract (Scale bar 300 nm), (D) selenium NPs prepared using selenous acid and *Punica granatum* seed extract (Scale bar 500 nm), (E) selenium NPs prepared using selenium tetrachloride and *Punica granatum* peel extract (Scale bar 500 nm), and (F) selenium NPs prepared using selenous acid and *Punica granatum* juice (Scale bar 500 nm), Figure S3: characteristics of iron NPs. Representative energy-dispersive X-ray analysis of iron NPs prepared with (A) *Punica granatum* peel extract using ferric chloride, (B) *Punica granatum* juice using ferric chloride, and (C) *Punica granatum* juice using ferrous sulphate, Figure S4: characteristics of selenium NPs. Representative energy-dispersive X-ray analysis of selenium NPs prepared with (A) *Punica granatum* peel extract using selenium chloride, (B) *Punica granatum* juice using selenous acid, and (C) *Punica granatum* juice using selenium chloride. Table S1: cytotoxicity of the phytoconstituents to the MCF-7 cell line.

## Sample Availability

Samples of the compounds are not available from the authors.
