# Supplementary material for: Green Synthesis of Gold, Iron and Selenium Nanoparticles Using Phytoconstituents: Preliminary Evaluation of Antioxidant and Biocompatibility Potential
Source: Molecules. 2022 Feb 16;27(4):1334. doi: 10.3390/molecules27041334 (PMC8875721; doi:10.3390/molecules27041334)
Supplement: Supplementary file 1 [file molecules-27-01334-s001.zip › molecules-1561296-supplementary.pdf]

Supplementary Materials

Green Synthesis of Gold, Iron and Selenium Nanoparticles Using Phytoconstituents: Preliminary Evaluation of Antioxidant and Biocompatibility Potential

Abeer Jabra Shnoudeh [1], Lana Qadumii [2], Malek Zihlif [3], Hamzeh J. Al-Ameer [3,4], Ruba Anwar Salou [2], Abdulmutalleb Yousef Jaber [1] and Islam Hamad [5,*]

[1] Department of Pharmaceutical Sciences, Faculty of Pharmacy, Philadelphia University, Amman 19392, Jordan; ashnoudeh@philadelphia.edu.jo (A.J.S.), ajaber@philadelphia.edu.jo (A.Y.J.)

[2] Department of Basic Sciences, Faculty of Science, Philadelphia University, Amman, 19392, Jordan; lqadumii@philadelphia.edu.jo (L.Q.); rsalou@philadelphia.edu.jo (R.A.S.)

[3] Department of Pharmacology, School of Medicine, The University of Jordan, Amman 11942, Jordan; m.zihlif@ju.edu.jo (M.Z.); h.alameer@aum.edu.jo (H.J.A.A.)

[4] Department of Biology and Biotechnology, Faculty of Science, American University of Madaba, Madaba 11821, Jordan

[5] Department of Pharmacy, Faculty of Health Sciences, American University of Madaba, Madaba 11821, Jordan

* Correspondence: i.hamad@aum.edu.jo; Tel: +962799585892

Supplementary Table 1. Cytotoxicity of the phytoconstituents to the MCF-7 cell line.

		400 mcg/ml	200 mcg/ml	100 mcg/ml	50 mcg/ml	25 mcg/ml	12.5 mcg/ml	6.25 mcg/ml	3.125 mcg/ml	1.5625 mcg/ml		
Punica granatum	Viability	1.134783	0.908696	0.989855	1.121739	1.097101	1.156522	0.982609	0.923913	1.154348	Viability	Punica granatum
	std	0.122975	0.037063	0.012467	0.165141	0.162073	0.056522	0.026087	0.041304	0.01087	std	
Pistachio	Viability	0.99453	1.014957	1.079772	1.052707	1.037037	1.014245	0.957265	0.878917	0.91453	Viability	Pistachio
	std	0.151709	0.019231	0.091811	0.092472	0.138272	0.043906	0.087233	0.029054	0.054505	std	
Ephedra	Viability	0.905858	1.09484	1.037657	0.956764	0.893305	1	1.008368	0.898187	0.951185	Viability	Ephedra
	std	0.031381	0.054393	0.016736	0.035565	0	0.075314	0.023254	0.054393	0.004184	std	

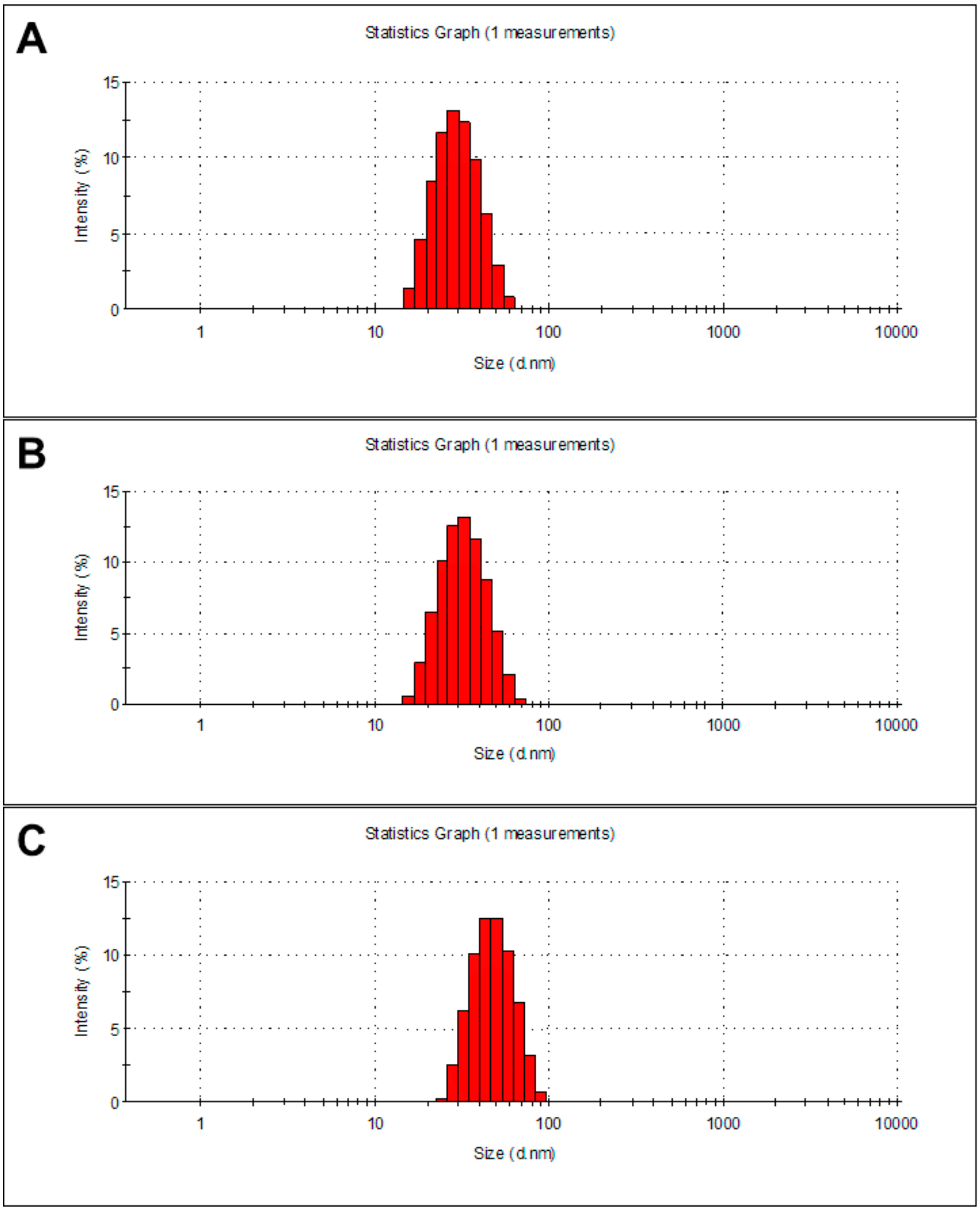

Supplementary figure 1. Histograms obtained from the Zetasizer for the (A) gold nanoparticles using ephedra extract, (B) iron nanoparticles using *Punica granatum* extract and (C) selenium nanoparticles using *Punica granatum* extract.

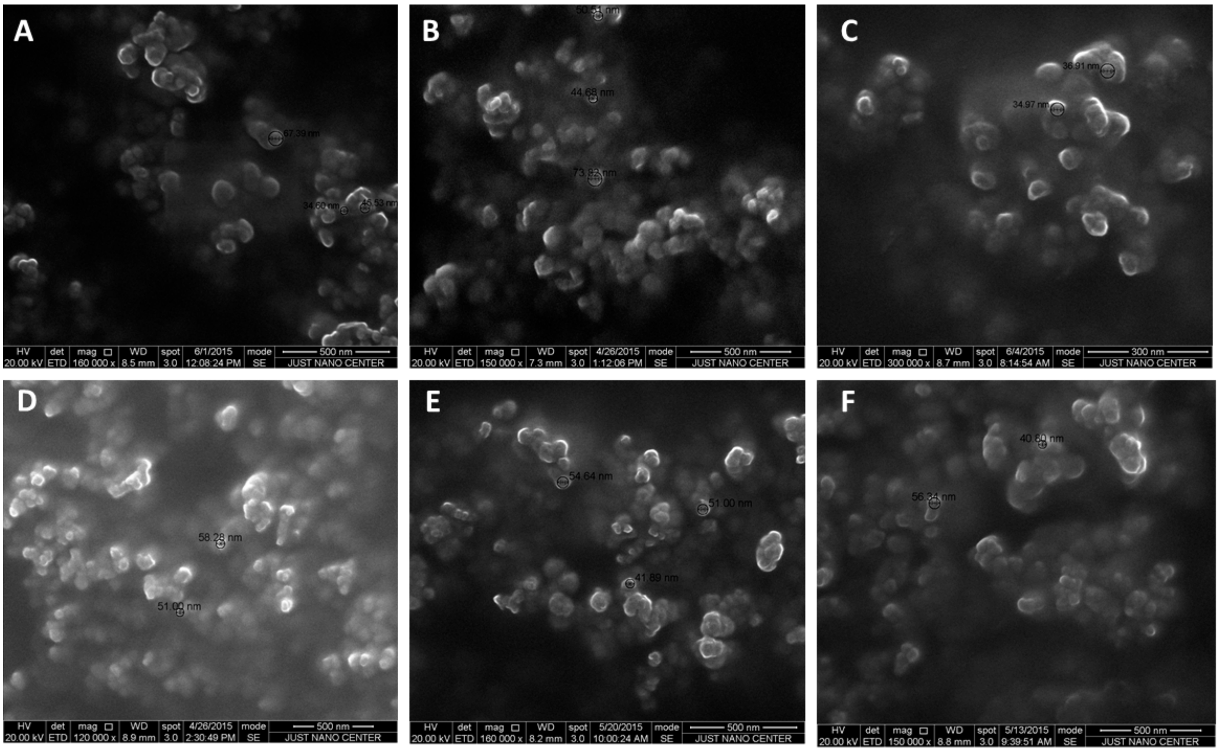

Supplementary figure 2. Scanning electron micrographs of prepared nanoparticles. Representative images A) iron nanoparticles prepared with *Punica granatum* seed extract using ferrous sulphate, B) iron nanoparticles prepared with *Punica granatum* seed extract using iron (III) perchlorate, C) selenium nanoparticles with *Punica granatum* seed extract using selenium tetrachloride, D) selenium nanoparticles with *Punica granatum* seed extract using selenous acid, E) selenium nanoparticles with *Punica granatum* peel extract using selenium tetrachloride, and F) selenium nanoparticles with *Punica granatum* juice using selenous acid.

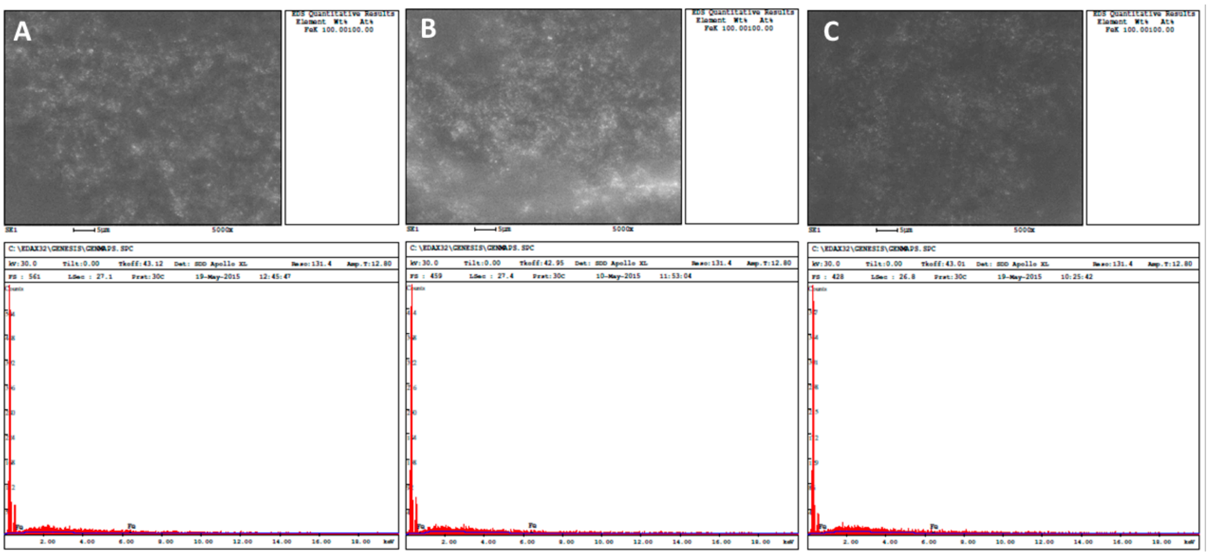

Supplementary figure 3. Characteristics of iron nanoparticles. Representative energy-dispersive X-ray analysis of iron nanoparticles prepared with A) *Punica granatum* peel extract using ferric chloride, B) *Punica granatum* juice using ferric chloride, and C) *Punica granatum* juice using ferrous sulphate.

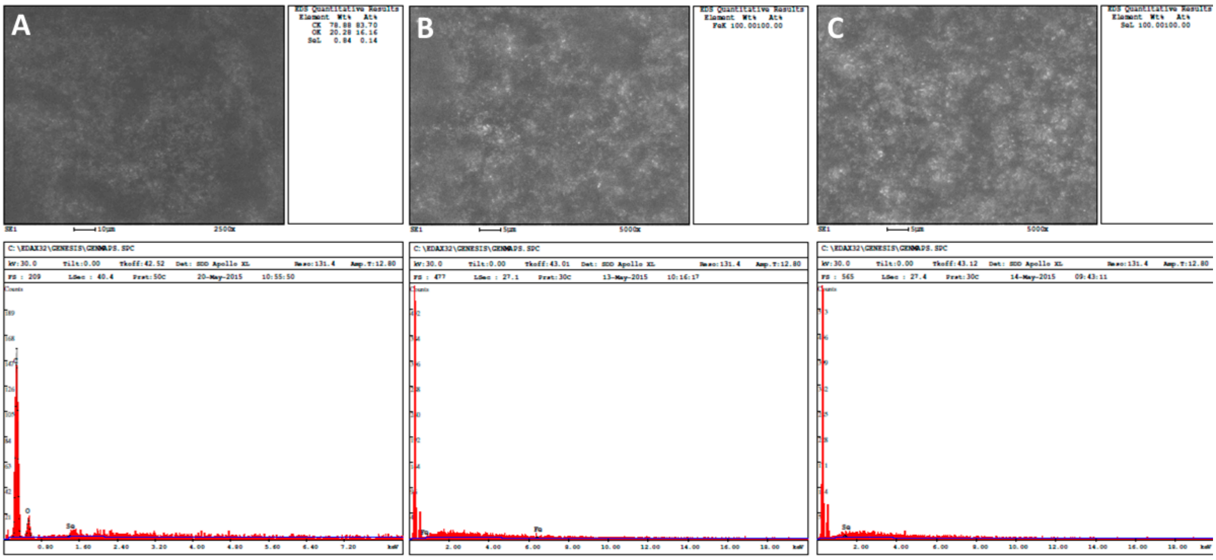

Supplementary figure 4. Characteristics of selenium nanoparticles. Representative energy-dispersive X-ray analysis of iron nanoparticles prepared with A) *Punica granatum* peel extract using selenium chloride, B) *Punica granatum* juice using selenous acid, and C) *Punica granatum* juice using selenium chloride.
